# Tolerance strategies revealed in tree peony (*Paeonia suffruticosa*; Paeoniaceae) ecotypes differentially adapted to desiccation

**DOI:** 10.1002/aps3.1191

**Published:** 2018-10-25

**Authors:** Lili Guo, Dalong Guo, Weilun Yin, Xiaogai Hou

**Affiliations:** ^1^ College of Agriculture Henan University of Science and Technology Luoyang 471023 People's Republic of China; ^2^ College of Forestry Henan University of Science and Technology Luoyang 471023 People's Republic of China; ^3^ College of Biological Sciences and Technology Beijing Forestry University Beijing 100083 People's Republic of China

**Keywords:** drought tolerance, *Paeonia suffruticosa*, Paeoniaceae, transcriptome analysis, tree peony

## Abstract

**Premise of the Study:**

Tree peony (*Paeonia suffruticosa*; Paeoniaceae) is well known for its ornamental value, edible oil, and medicinal properties. However, its growing area has been limited by drought that has been exacerbated by global climate change.

**Methods:**

Gene expression profiles of a drought‐tolerant cultivar and a drought‐sensitive cultivar during dehydration and rehydration were investigated by transcriptome analysis. Expression patterns of unigenes related to drought and recovery response and unrelated to either cultivar were classified by hierarchical clustering and real‐time quantitative PCR (qPCR).

**Results:**

A total of 81,725 unigenes with a mean length of 762 nucleotides that may play roles in drought response were identified. Unigenes were characterized as being involved in lipid transport metabolism, proline metabolism, and photosynthesis. In addition, plant hormone signaling pathway genes were also characterized as potentially being involved in drought response. Expression patterns of the 20 drought‐responsive unigenes verified by qPCR showed a differential expression pattern under either the drought or recovery treatment.

**Discussion:**

This is the first report to identify and verify unigenes of tree peonies with differing water sensitivity during dehydration and rehydration. This study offers a valuable resource for candidate genes involved in drought and provides insight into the breeding of drought‐resistant tree peony cultivars.

Tree peony (*Paeonia suffruticosa* Andrews; Paeoniaceae) has been cultivated for more than 1600 years in China, and there are approximately 2000 tree peony cultivars worldwide (Wang, 1997). The first written description of this genus was in 200 BC as a medicinal plant. In the fifth century, with the selection of multiple flower shapes and colors, it became known as an ornamental plant (Li et al., 2011). Studies have shown that the seed oil of tree peony contains abundant unsaturated fatty acids that are beneficial to human health (Sarker et al., 1999; Su et al., 2016). Owing to its multiple uses, the peony genus has spread through Asia, to the Mediterranean, Caucasus Mountains, the mountainous regions of Europe, the United States, and Australia (Rogers, 1995).

The greatest number of cultivated varieties and the largest distribution area of tree peony are found in central China, which has long been characterized by an arid climate. Nevertheless, severe water deficiency stress can limit the cultivation area, lead to smaller leaves and flowers, inhibit the synthesis of organic substances and flower pigments, and reduce the ornamental value and seed yield of tree peony (Li et al., 2011). Recent studies, however, have mainly focused on oil extraction (Chen et al., 2016a, b; Cui et al., 2016; Han et al., 2016). In addition, efficient protocols for the micropropagation of tree peony and the effects of different medium compositions and exogenous hormones on the browning of tree peony callus in tissue culture were recently investigated (Wen et al., 2016; Zhou et al., 2016a). Surprisingly, however, the desiccation tolerance strategies in tree peony cultivars have not yet been investigated.

Transcriptome analysis that uses deep sequencing technology now permits large‐scale gene expression detection in the absence of a reference genome. Although there have been several investigations of transcriptome sequencing of tree peony (Gai et al., 2012; Zhou et al., 2013; Zhang et al., 2014, 2015; Zhao et al., 2014; Barghini et al., 2015; Li et al., 2015; Shi et al., 2015; Gao et al., 2016; Wang et al., 2016b), studies of drought‐responsive differential expression genes in tree peonies have not yet been reported in the literature. Two separate studies of reference gene selection in tree peony—one in plants with different flower colors and another during flower development—were recently reported (Li et al., 2016; Zhou et al., 2016b).

Screening of drought‐tolerant tree peony cultivars revealed that ‘Luo Yang Hong’ (LYH) is tolerant to drought, whereas ‘Wu Long Peng Sheng’ (WLPS) is tolerant to flooding (Kong et al., 2011), making the two cultivars ideal study material for investigating mechanisms of drought response in plants. With a view toward improving plant structure, perfecting bloom quality, and mitigating damage from desiccation, this study used LYH and WLPS with their contrasting water sensitivity to characterize unigenes during dehydration and rehydration to explore the complex mechanisms of drought response networks.

## Materials and Methods

### Plant material treatment and sample collection

Four‐year‐old LYH and WLPS seedlings were cultured in pots using soil collected from the Luoyang National Peony Garden (Luoyang, China). The pots were buried deep in the ground from October until May to avoid freezing injury. The pots were then dug out and irrigated once every two days and cultured under natural conditions before they were used for the water deficiency treatments. Five individuals were used per treatment. The drought treatment (DR) was initiated by tap water irrigation until the soil moisture content reached 80%, after which plants were dehydrated naturally for seven days until the leaves had severely wilted. For the rehydration treatment (RE), the tree peonies were re‐watered until the soil moisture content again reached 80%, after which they were cultured for one more day to let the leaf blades completely unfold. Tree peonies cultured in pots with a constant soil moisture content of 80% served as the control treatment (CK). The soil moisture content was measured by gravimetric methods (Bao, 2000). For all three treatments, the third and fourth leaves were sampled and immediately frozen in liquid nitrogen and stored at −80°C.

### RNA extraction, cDNA library construction, and sequencing

The leaves sampled from the three different treatments (DR, RE, and CK) were assigned to six independent pools. Total RNA was extracted using the modified cetyltrimethylammonium bromide (CTAB) method (Gambino et al., 2008). The integrity of RNA was examined by an Agilent 2100 Bioanalyzer (Agilent Technologies, Santa Clara, California, USA). An in‐house library preparation method was used for mRNA sequencing, using fragment sizes of 200 bp. Libraries were quantified by an Agilent Bioanalyzer and qualified by the ABI StepOnePlus Real‐Time PCR System (Thermo‐Fisher Scientific, Waltham, Massachusetts, USA) during the quality control steps. Libraries were sequenced using the Illumina HiSeq 2000 (Illumina, Shenzen, Guangzhou, China) at the Beijing Genomics Institute (BGI). Each library was run on a separate lane of the HiSeq. The cDNA library was deposited in the National Center for Biotechnology Information (NCBI) Transcriptome Shotgun Assembly database (BioSample accession no. SRS1180651).

### De novo assembly and protein‐coding region prediction

Reads with adapters, unknown nucleotides larger than 5%, and low‐quality reads (bases quality ≤10) were discarded. Only reads longer than 90 bp were used for assembly. Reads from all treatments and/or cultivars were assembled together by Trinity 3.4 (Grabherr et al., 2011; open source code publicly available at http://TrinityRNASeq.sourceforge.net). Because a reference genome is not available for tree peony, reads were mapped to the assembled unigene set.

Unigenes were first aligned by BLASTX (*E*‐value <0.00001) to protein databases in the following order of NCBI's nonredundant protein database (nr), Swiss‐Prot, Kyoto Encyclopedia of Genes and Genomes (KEGG), and NCBI's Clusters of Orthologous Groups (COG) database. Unigenes aligned to a higher‐priority database were not aligned to lower‐priority databases. The best alignment results were used to decide sequence direction of unigenes. When a unigene was not aligned with any of the above databases, ESTScan was used to determine its sequence direction (Iseli et al., 1999).

Proteins with the highest ranks in the BLAST results were used to decide the coding region sequences of unigenes, then the coding region sequences were translated into amino sequences with the standard codon table. Unigenes that could not be aligned to any database were scanned by ESTScan (Iseli et al., 1999), producing nucleotide sequence (5′–3′) direction and amino sequence of the predicted coding region.

### Gene ontology classification and metabolic pathway analysis

Unigene functional classification and annotation was performed by WEGO (http://wego.genomics.org.cn/) (Ye et al., 2006). Gene function in cellular processes and gene products during metabolism process were analyzed by KEGG (http://www.genome.jp/kegg) (Kanehisa et al., 2008).

### Real‐time quantitative PCR (qPCR) verification analysis

Twenty dehydrin‐related unigenes were selected for the assessment of expression profiles. Total RNA was converted into single‐stranded cDNA using an M‐MLV reverse transcriptase (Promega Corporation, Madison, Wisconsin, USA). The ABI StepOnePlus Real‐Time PCR System (Thermo‐Fisher Scientific) was utilized to perform the expression profile verification. The reaction was carried out as described in our previous publication (Pang et al., 2015), using three biological replicates per sample. The relative gene expression levels of the selected unigenes were normalized to 18S rRNA and calculated using the 2‐ΔΔCt method (Livak and Schmittgen, 2001). The primer sequences are shown in Table 1.

**Table 1:** Primer sequences used for real‐time quantitative PCR (qPCR)

Gene	Forward primers (5′–3′)	Reverse primers (5′–3′)
Unigene8873_All_LYH‐DR	ACAAGACCCCCGAGCTTTTT	CATATGCATCCGTCTGGCGA
CL8710.Contig2_All_LYH‐DR	GACCCTCCCAAACAGTCGTC	TGTTCGTCGGTGTCTGATCC
Unigene5006_All_LYH‐DR	CGGCTTATCGTATGCGTGGT	GCAGCTCCGTTCCGAGTTTA
Unigene16234_All_LYH‐DR	AGGCCAAAACAGGGGAACAC	CCTTTGACACAGCCGAGGAA
CL2427.Contig2_All_LYH‐DR	TTGGCGAGATCGTCACTTCC	AAGACGGCGTCGGTTCTATC
Unigene383_All_LYH‐DR	ACGGGCGAAGACGACAATAA	CACTACTGGTTGTGCGGCAT
Unigene18390_All_LYH‐DR	CGAGTGCCAAAGGGAGAGTT	GCAGACTCGTCGTCTGACTT
CL9864.Contig2_All_LYH‐DR	CGCACTCGTCATGTCCTACTT	GCACAGCTTACGCGACTAAC
Unigene1202_All_LYH‐DR	ACTAATATCGGCGGGGAGGA	CCCTCCTCACCTCTACCCTC
Unigene1395_All_LYH‐DR	ACTGCTTGTCTCAAGCTCACTT	TCATCGGTGATCGTGGAAGC
CL4531.Contig1_All_LYH‐RE	CCGACGTGCTCTGACATGAA	AAGGTGCAGAACCCAAAGGT
CL154.Contig2_All_LYH‐RE	CCAGACCCAGCAACTCTGTT	CGCTGGTCACCATTTTGCTC
Unigene4037_All_LYH‐RE	ATGGCTTAACAAGCACCCGA	TTTACGGGCCTGTGCAAGAT
Unigene25204_All_LYH‐RE	CGCCTCACACCAAAAGTCAAG	CTTTCAACAACAGGGCACGG
CL3906.Contig3_All_LYH‐RE	ATGCCGAACCAACTACACGA	TCACCGCAGAGCATAACTGG
CL10838.Contig2_All_LYH‐RE	GCGGCAACTACGTCTTTTGA	CGAGAGCGAAGAGAGCATGT
Unigene15264_All_LYH‐RE	AGGCAAGTACGTGGGAGGTA	CCCAGAACATCTCCGACACG
Unigene32639_All_LYH‐RE	AAGTAGAGCCCAAGCAGCAT	CGTATCCAGGCGGAGCTTTT
CL7346.Contig2_All_LYH‐RE	AACAGTACTCCTCGTCCGGT	GGAGTCCATACCGATGTGCC
CL7474.Contig3_All_LYH‐RE	GCATGTCGACGATGAACACG	TTCGCCCCTCTTGTCAATCC

## Results

### Sequencing output statistics, assembly metrics, and protein‐coding region classification

The total number of clean nucleotides generated from the six libraries of the two tree peony cultivars (LYH and WLPS) by three treatments (CK, DR, and RE) exceeded 4.6 Gbp. The number of clean reads of LYH‐CK, LYH‐DR, LYH‐RE, WLPS‐CK, WLPS‐DR, and WLPS‐RE were 54, 52, 52, 52, 51, and 52 million, respectively. The Q20 values exceeded 97%, and the GC contents were approximately 46% of all samples, which meant that the sequencing data were robust for further analysis. After assembling all sequences from all samples, 81,725 unigenes were obtained. Their aggregate length was 62,310,011 nucleotides, with a mean length of 762 nucleotides.

**Figure 1:**
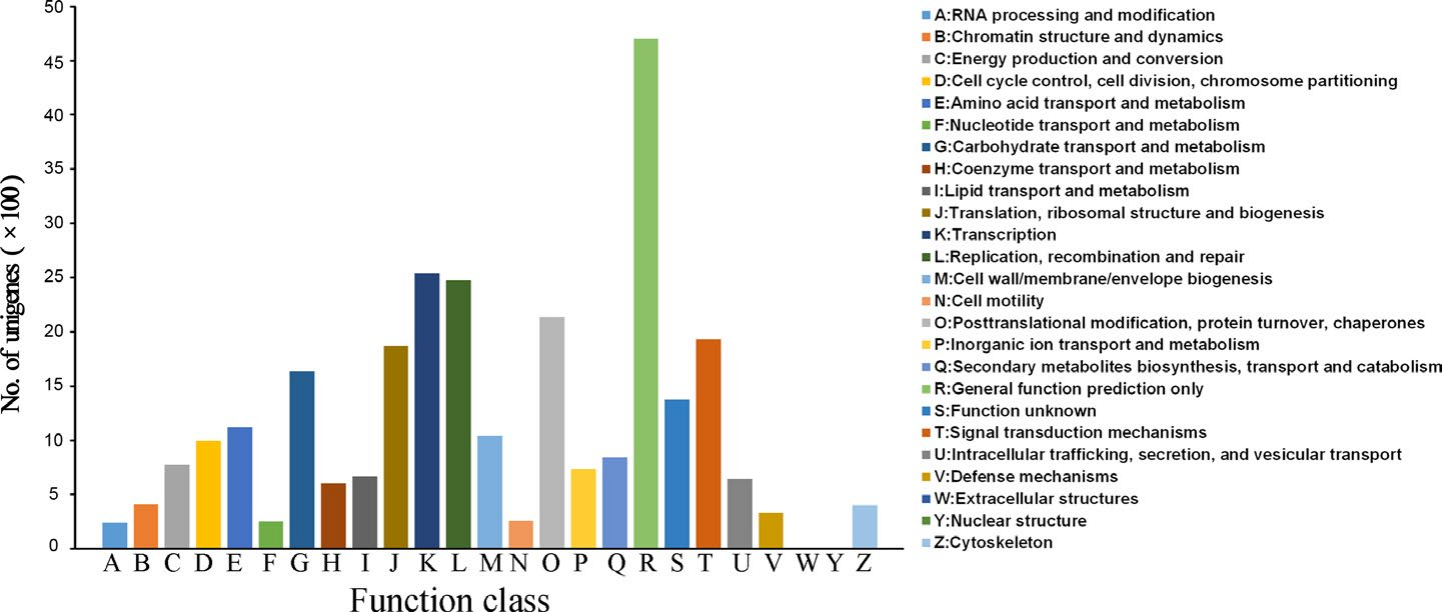
Clusters of Orthologous Groups (COG) function classification of all unigenes. The letters on the *x*‐axis indicate the COG categories listed to the right of the histogram.

A total of 41,808 protein‐coding regions from the unigenes were translated into amino sequences. Detailed information of their length distributions is given in Appendix 1. The COG analysis showed that 14,768 unigenes were assigned to 25 classifications. The largest category was ‘General function prediction only’; ‘Transcription,’ ‘Replication, recombination, and repair,’ ‘Post transcriptional modification, protein turnover, chaperones,’ and ‘Signal transduction merchanism’ were comparatively high; and ‘RNA processing and modification’ had the smallest number of responding unigenes (Fig. 1).

### Functional annotation of the unigenes

**Figure 2:**
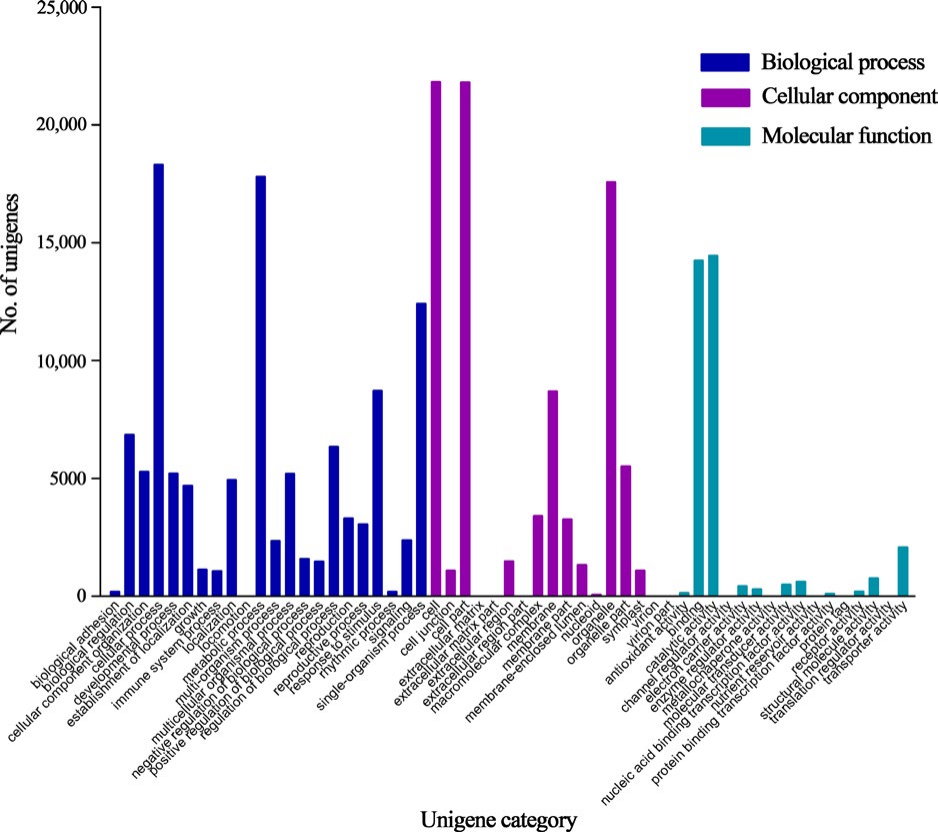
Functional category distribution of assembled unigenes in tree peony. The results were summarized in three main categories: biological process, cellular component, and molecular function.

A total of 43,977 unigenes were successfully allocated to the three main gene ontology categories: biological process, molecular function, and cellular component (Fig. 2). The biological process category contained 22 classes subsumed under five larger groups: cellular process (18,310), metabolic process (17,809), single‐organism process (12,415), stimulus (8451), and biological regulation (6850). The cellular components category consisted of 17 classes, dominated by cell (21,823), cell part (21,822), and organelle (17,580). The molecular function category consisted of 16 classes, for which catalytic activity (14,450), binding (14,248), and transporter activity (2069) were the largest groups.

Significantly enriched gene ontology terms with a larger cluster frequency (i.e., ≥9%) were identified. For the biological process category, the oxygen‐containing compound and oxidation‐reduction process were more common not only among treatments (CK, DR, and RE) within a cultivar but also between the cultivars (LYH and WLPS) for a given treatment. The unigenes responded to stimuli, including abiotic, endogenous, biotic, and chemical stimuli that were identified extensively in LYH and WLPS. Unigenes’ response to stress was detected only in the CK vs. RE treatments of LYH (Appendix 2). For the molecular function category, unigenes were identified in DR vs. RE of LYH, while oxidoreductase and catalytic activity were largely detected both between cultivars and among the treatments. None of the other candidate molecular functions were identified, except the above two functions between LYH and WLPS (Appendix 3). For the cellular component category, the membrane, cell periphery, plasma membrane, and extracellular region were extensively detected in the LYH and WLPS treatments separately. Within the same treatment, however, none of the unigenes responded to the cellular component between the cultivars (LYH and WLPS) apart from two exceptions: the membrane detected in DR and the extracellular region identified in RE (Appendix 4).

### Identification of dehydration‐ and rehydration‐responsive unigenes

**Figure 3:**
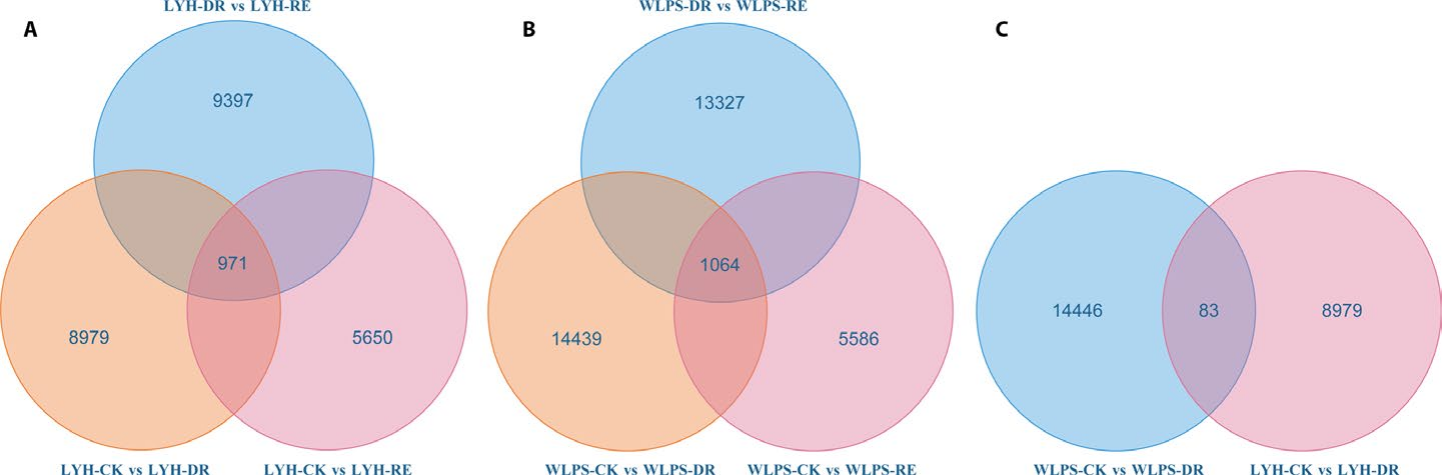
Venn diagram of unigenes with annotation from the cultivars ‘Luo Yang Hong’ (LYH) and ‘Wu Long Peng Sheng’ (WLPS). The Venn diagram shows the overlapping unigene response to both drought (DR) and rehydration (RE) in LYH (A) and WLPS (B), and the overlapping unigenes related to each cultivar (C).

**Figure 4:**
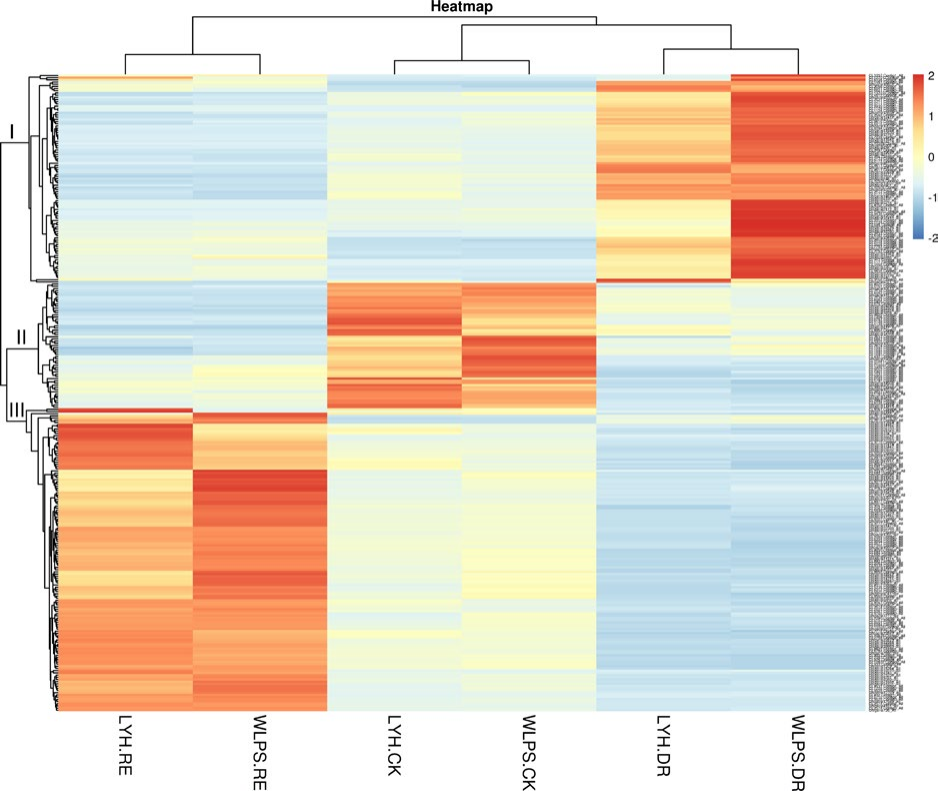
Hierarchical clustering of three accumulated water phases for the cultivars ‘Luo Yang Hong’ (LYH) and ‘Wu Long Peng Sheng’ (WLPS)*. x*‐axis: sample information. A dendrogram based on the analysis of the 290 drought‐responsive unigenes is shown on the left of the figure, while a dendrogram based on a clustering analysis of the samples is shown above the figure. Colors indicated the unigene expression levels, with darker colors signifying higher levels of expression. Red indicates unigenes that were upregulated, whereas blue indicates unigenes that were downregulated.

A total of 971 drought‐responsive unigenes in LYH were identified by comparison of LYH‐CK vs. LYH‐DR (8979), LYH‐CK vs. LYH‐RE (5650), and LYH‐DR vs. LYH‐RE (9397) (Fig. 3A), whereas 1064 drought‐responsive unigenes in WLPS were identified by comparison of WLPS‐CK vs. WLPS‐DR (14,446), WLPS‐CK vs. WLPS‐RE (5593), and WLPS‐DR vs. WLPS‐RE (13,327) (Fig. 3B). Further comparison identified 373 unigenes in both LYH and WLPS. Excluding the 83 unigenes accessed by the comparison of LYH‐CK vs. LYH‐DR and WLPS‐CK vs. WLPS‐DR (Fig. 3C), 290 unigenes lacking any relationship with genotype yet responding to dehydration and rehydration were detected. Hierarchical clustering of LYH‐CK, LYH‐DR, LYH‐RE, WLPS‐CK, WLPS‐DR, and WLPS‐RE indicated that CK, DR, and RE were clustered together, revealing dehydration‐ and rehydration‐responsive unigenes (Fig. 4). We note that our differential expression results are preliminary, as our experiments lack replication. Follow‐up experiments will be needed to fully validate our observations.

### Pathway analysis using the KEGG database

BLAST analysis of 81,725 unigenes against the KEGG database was performed to analyze gene products during metabolism processes and related gene functions in cellular processes. A total of 23,518 unigenes were involved in 128 KEGG pathways. More than one‐fifth (21.77%) were related to metabolic pathways, whereas 11.82% were related to the biosynthesis of secondary metabolites, 5.51% were related to plant–pathogen interaction, and 4.69% were related to plant hormone signal transduction (Table 2).

**Table 2:** Kyoto Encyclopedia of Genes and Genomes (KEGG) pathway statistics

Pathway	No. of genes with pathway annotation (%)^a^	Pathway ID
Metabolic pathways	5121 (21.77%)	ko01100
Biosynthesis of secondary metabolites	2781 (11.82%)	ko01110
Plant–pathogen interaction	1296 (5.51%)	ko04626
Plant hormone signal transduction	1103 (4.69%)	ko04075
Spliceosome	972 (4.13%)	ko03040
RNA transport	953 (4.05%)	ko03013
RNA degradation	746 (3.17%)	ko03018
Ribosome biogenesis in eukaryotes	745 (3.17%)	ko03008
Protein processing in endoplasmic reticulum	627 (2.67%)	ko04141
Ribosome	625 (2.66%)	ko03010
Endocytosis	613 (2.61%)	ko04144
Starch and sucrose metabolism	541 (2.3%)	ko00500
Glycerophospholipid metabolism	538 (2.29%)	ko00564
Pyrimidine metabolism	510 (2.17%)	ko00240
mRNA surveillance pathway	505 (2.15%)	ko03015
Purine metabolism	503 (2.14%)	ko00230
Ubiquitin‐mediated proteolysis	433 (1.84%)	ko04120
Phenylpropanoid biosynthesis	380 (1.62%)	ko00940
Ether lipid metabolism	366 (1.56%)	ko00565
Zeatin biosynthesis	310 (1.32%)	ko00908
Glycolysis/gluconeogenesis	310 (1.32%)	ko00010
Oxidative phosphorylation	300 (1.28%)	ko00190
Terpenoid backbone biosynthesis	285 (1.21%)	ko00900
ABC transporters	279 (1.19%)	ko02010
RNA polymerase	259 (1.1%)	ko03020
Pentose and glucuronate interconversions	245 (1.04%)	ko00040
Amino sugar and nucleotide sugar metabolism	244 (1.04%)	ko00520
Homologous recombination	239 (1.02%)	ko03440

a The total number of genes was 23,518.

The KEGG pathways of unigenes with annotation for the two cultivars (LYH vs. WLPS) within the same treatment and among treatments (CK, DR, and RE) within the same cultivar were also analyzed. The metabolic pathways, biosynthesis of secondary metabolites, plant–pathogen interactions, and plant hormone signal transduction had similar percentages in all pathways identified. Abscisic acid, jasmonic acid, ethylene, brassinosteroids, salicylic acid, gibberellins, cytokinin, and auxin signaling pathways were all detected in this study. It is interesting to note that when analyzing the pathway between LYH and WLPS, the plant hormone signal transduction pathway was not detected in CK and RE (Table 3). LYH and WLPS exhibited higher plant–pathogen interaction after dehydration than the control, which might be caused by plant interaction with a pathogen such as arbuscular mycorrhizal fungi. However, it was unclear why a plant–pathogen interaction apparently went undetected after rehydration.

**Table 3:** Pathway analysis of unigenes with pathway annotation (*P* ≤ 0.05).^a^

Unigene comparison	Metabolic pathways	Biosynthesis of secondary metabolites	Plant–pathogen interaction	Plant hormone signal transduction
LYH‐CK vs. LYH‐DR	985/3972 = 24.8	622/3972 = 15.66	317/3972 = 7.98	260/3972 = 6.55
LYH‐CK vs. LYH‐RE	574/2366 = 24.26	378/2366 = 15.98	179/2366 = 7.57	170/2366 = 7.19
LYH‐DR vs. LYH‐RE	1131/4142 = 27.31	697/4142 = 16.83	355/4142 = 8.57	331/4142 = 7.99
WLPS‐CK vs. WLPS‐DR	1430/5905 = 24.22	838/5905 = 14.19	411/5905 = 6.96	368/5905 = 6.23
WLPS‐CK vs. WLPS‐RE	567/2234 = 25.38	334/2234 = 14.95	172/2234 = 7.7	159/2234 = 7.12
WLPS‐DR vs. WLPS‐RE	1455/5503 = 26.44	841/5503 = 15.28	416/5503 = 7.56	383/5503 = 6.96
LYH‐CK vs. WLPS‐CK	652/2332 = 27.96	409/2332 = 17.54	170/2332 = 7.29	—
LYH‐DR vs. WLPS‐DR	835/2843 = 29.37	507/2843 = 17.83	229/2843 = 8.05	160/2843 = 5.63
LYH‐RE vs. WLPS‐RE	596/2011 = 29.64	348/2011 = 17.3	—	—

a Values are number/total = percentage.

### Unigene validation by qPCR

To validate the expression profiling of the dehydrin‐responsive unigenes, 20 genes predicted to participate in the dehydrin response pathway were selected (1) to determine their relative expression in the dehydration (DR), rehydration (RE), and nontreatment of tree peony (CK) and (2) to validate the transcriptome sequencing results. Abundance of the target genes was normalized relative to the abundance of 18S RNA; the Ct values (i.e., the number of cycles corresponding to the inflection point from baseline to exponential growth) of 18S rRNA for all samples ranged from 24.0 to 26.0. The results of the qPCR verification showed a differential expression pattern under both the DR and RE treatments. Dehydrin Xero 2‐like was significantly upregulated after dehydration but then downregulated during rehydration of tree peony seedlings (Table 4).

**Table 4:** Real‐time quantitative PCR (qPCR) validation of 20 unigenes in three tree peony treatment groups: dehydration, rehydration, and a control group

Gene ID	Nr annotation	2^‐ΔΔ CT^	Log2 (DR_FPKM or RE_FPKM/CK_FPKM)
Unigene8873_All_LYH‐DR	Dehydrin [*Paeonia suffruticosa*]	5.3658	4.4933
CL8710.Contig2_All_LYH‐DR	Ethylene response factor 11 [*Actinidia deliciosa*]	−0.1155	−1.4333
Unigene5006_All_LYH‐DR	Ethylene responsive transcription factor 1A [*Prunus salicina*]	5.0722	3.5543
Unigene16234_All_LYH‐DR	Ethylene‐responsive transcription factor 1B, putative [*Ricinus communis*]	9.1154	4.4226
CL2427.Contig2_All_LYH‐DR	GDSL esterase/lipase EXL3 [*Vitis vinifera*]	−2.7565	−3.0232
Unigene383_All_LYH‐DR	GDSL esterase/lipase 1 [*Vitis vinifera*]	4.5329	2.5685
Unigene18390_All_LYH‐DR	RING‐H2 finger protein ATL60‐like [*Vitis vinifera*]	5.7046	3.3146
CL9864.Contig2_All_LYH‐DR	RING‐H2 finger protein ATL78 [*Vitis vinifera*]	−1.6611	−2.444
Unigene1202_All_LYH‐DR	RING‐H2 zinc finger protein RHA4a [*Vitis vinifera*]	−3.5098	−4.4392
Unigene1395_All_LYH‐DR	Transcription factor bHLH135 [*Vitis vinifera*]	−0.0729	−2.4395
CL4531.Contig1_All_LYH‐RE	Transcription factor bHLH63‐like [*Vitis vinifera*]	−1.4411	−1.5683
CL154.Contig2_All_LYH‐RE	NAC domain‐containing protein 72 [*Vitis vinifera*]	−0.3531	−2.7657
Unigene4037_All_LYH‐RE	Zinc finger CCCH domain‐containing protein 53‐like [*Glycine max*]	−1.0864	−2.9649
Unigene25204_All_LYH‐RE	MYBF1 [*Vitis vinifera*]	−0.5343	−1.4983
CL3906.Contig3_All_LYH‐RE	Uncharacterized calcium‐binding protein At1g02270 [*Vitis vinifera*]	6.7048	4.9768
CL10838.Contig2_All_LYH‐RE	Universal stress protein A‐like protein [*Vitis vinifera*]	9.8757	3.6137
Unigene15264_All_LYH‐RE	TIR‐NBS type disease resistance protein [*Populus trichocarpa*]	7.0632	5.5444
Unigene32639_All_LYH‐RE	Heavy metal–associated isoprenylated plant protein 26‐like [*Fragaria vesca* subsp. *vesca*]	13.1831	3.3610
CL7346.Contig2_All_LYH‐RE	Glutamate dehydrogenase, putative [*Ricinus communis*]	14.1086	7.3002
CL7474.Contig3_All_LYH‐RE	17.9 kDa class II heat shock protein isoform 1 [*Vitis vinifera*]	−1.4672	−3.6081

Note

CK = control treatment; DR = drought treatment; FPKM = fragments per kilobase of transcript per million mapped reads; Nr = National Center for Biotechnology Information nonredundant protein database; RE = rehydration treatment.

## Discussion

Drought stress is one of the main abiotic stresses, and it may alter plant growth, metabolism, and yield (Ajithkumar and Panneerselvam, 2014). In tree peonies cultivated in central and northwestern China, water deficiency is a common problem. This drought stress limits the growth of leaves and flowers, inhibits the synthesis of organic compounds and anthocyanin, and reduces seed yield (Li et al., 2011). Plants that receive drought signals initiate a range of physiological, morphological, and biochemical defense responses at both the cellular and molecular level (Verslues et al., 2006). An overexpression of genes in response to drought stress could alleviate drought‐induced damage while promoting plant growth. Drought tolerance strategies, as revealed by transcriptome sequencing in poplar (Barghini et al., 2015) and sorghum (Fracasso et al., 2016), have uncovered a number of drought‐responsive genes. To diminish the damage caused by water deficiency in tree peony, two cultivars with contrasting water sensitivity were selected for unigene characterization to investigate the molecular mechanisms driving their drought response.

Plants can adapt to desiccation stresses and stay alive by alternating the accumulation of osmolytes (Parida et al., 2007). Proline, one of the most important osmolytes, is quickly accumulated and involved in the plant response to dehydration to maintain a cellular balance of water content and turgor potential (Vendruscolo et al., 2007). A previous study showed that proline accumulates after dehydration and then decreases to the initial level after rehydration in both LYH and WLPS tree peony cultivars (Li et al., 2014). Proline variation during dehydration and rehydration corresponded to transcriptome analysis and was consistent with that reported in previous publications (Gechev et al., 2012; Hossain et al., 2016). In the present study, numerous unigenes related to the proline metabolism process, including proline‐rich proteins (Unigene8963_All, Unigene11447_All), proline transporters (Unigene11945_All), and an osmotic precursor (CL5318.Contig1_All) were identified in LYH and WLPS under the dehydration and rehydration treatments.

One of the most harmful effects of drought stress is increased production of reactive oxygen species (ROS) (Miller et al., 2010; Bartwal et al., 2016). When plants suffer drought stress, active oxygen metabolism is strengthened, which generates a large amount of O_2_^−^, H_2_O_2_, and OH^−^ (Bian and Jiang, 2009). However, plants possess an evolved antioxidant defense system that enables them to maintain ROS at a low quantity to protect cells from excessive and permanent oxidative damage. Therefore, the ability of plants to clear the active oxygen can reflect their ability to tolerate stress (Hossain et al., 2016). Induction of the antioxidant enzymes’ expression level makes the antioxidant system an efficient mechanism to control ROS accumulation, both temporally and spatially (Hossain et al., 2016). These redox‐sensitive proteins may be oxidized by ROS directly or indirectly via non‐enzymatic compounds, such as glutathione (GSH), which are major players in redox signaling when antioxidants are involved (Shao et al., 2008; Klumpen et al., 2016; Thangamani et al., 2016). The unigene corresponding to glutamate (CL7346.Contig2_All) displayed a different responsive pattern in tree peony when exposed to dehydration and rehydration.

Large pigment–protein complexes are the most significant factors that function during photosynthesis (Qin et al., 2015). Light‐harvesting complex stress‐related proteins catalyze excess energy dissipation in photosystems I (PSI) and II (PSII) (Pinnola et al., 2015). The unigenes involved in photosynthesis include 14 subunit complexes in PSI and another 14 subunit complexes in PSII, which were examined in the present study. Specifically, the chlorophyll a/b‐binding protein that encodes the light‐harvesting complex (LHC), which captures sunlight and transfers the excitation energy to the core in higher plants, was obtained. Four unique subunits (PsaG, PsaH, PsaN, and PsaO) of the PSI‐LHCI super complex in higher plants were detected in the present study. As a member of the ROS, H_2_O_2_ and its production occur in the chloroplasts, peroxisomes, and mitochondria of plants. Drought‐treated plants had a significantly increased ROS content and diminished operating and maximum efficiencies of PSII photochemistry (Ryan et al., 2014). Reduced photosynthetic pigment contents resulting from drought stress might decrease ROS formation by regulating chlorophyll synthesis and other components of the photosynthetic machinery.

ROS generation is considered to be closely related to lipid peroxidation under drought stress (Ryan et al., 2014; Uzilday et al., 2014). For example, lipid peroxidation analysis showed that transgenic *Agrostis stolonifera* L. root exhibited less cellular damage when compared with the wild type under drought stress conditions (Xu et al., 2016). The alleviation of adverse effects of drought stress is partially attributable to an increased antioxidant ability and decreased lipid peroxidation induced by early ROS accumulation (Xing et al., 2016). Tobacco plants treated with low and moderate levels of riboflavin accumulated higher levels of ROS and lipid peroxide with enhanced drought tolerance (Deng et al., 2014). In the present study, 666 unigenes involved in lipid transport and metabolism were identified according to the COG classification. This clearly illustrates the close relationship between lipid peroxidation and the drought stress response in plants.

Calcium mobilization is one of the downstream events modulated by H_2_O_2_ (Neill et al., 2002). The calcium ion (Ca^2+^) functions as a secondary messenger in modulating diverse physiological processes that are important for stress adaptation in plants. Both Ca^2+^ and Ca^2+^/calmodulin (CaM)–binding protein and transcription factors have been identified, and their functional analysis suggests that they play key roles in plant stress signaling pathways (Reddy et al., 2011). Previous studies have indicated that drought stress activates ABA‐dependent and ABA‐independent gene expression (Yoshida et al., 2014). The *cis*‐regulatory element ABA‐responsive element (ABRE) (CACGTG [T/C/G]) and their coupling elements ([C/A]ACGCG[T/C/G]) in the upstream region were observed in the upregulated genes (Kaplan et al., 2006). Hence, it was concluded that for some specific Ca^2+^ transients, ABREs function as Ca^2+^‐responsive *cis*‐regulatory elements (Reddy et al., 2011).

ABRE and calcium‐dependent protein kinase (CDPK) have been found to be related to drought stress in other plant species (Yoshida et al., 2010; Zou et al., 2010). In the present study, ABREs (CL3759.Contig1_All and CL3759.Contig2_All) and CDPKs (unigene21495_All, unigene21823_All, CL5185.Contig3_All, and CL3906.Contig3_All) were identified. In addition, transcript accumulation of the myeloblastosis (MYB) transcription factor, the APETALA2/Ethylene Responsive Factor (AP2/ERF), the NAM, ATAF, AND CUC (NAC) transcription factor, the basic helix‐loop‐helix (bHLH) protein, and the zinc RING finger protein (RING‐H) were all identified after desiccation stress, which agrees perfectly with ABA accumulation (Nakashima and Yamaguchi‐Shinozaki, 2013; Furlan et al., 2014). Further studies are required to reveal their mechanisms of regulating drought resistance in tree peonies.

Heat stress can trigger the higher expression of heat‐shock proteins (HSPs), which might coordinate with other stress‐response mechanisms to mitigate cellular damage and re‐establish cellular homeostasis (Wang et al., 2004). Copper applied to tree peony revealed an increase in dehydration‐responsive element–binding (DREB) protein (Wang et al., 2016a). In the present study, we identified one class II HSP isoform 1 (CL7474.Contig3_All) and one heavy metal–associated isoprenylated plant protein (HIPP) (Unigene32639_All), both of which were unrelated to genotype but responsive to dehydration and rehydration. Regulation of HSP and HIPP by dehydration and rehydration in tree peony illustrates the synergistic interaction of drought with other stress‐response mechanisms to alleviate cellular damage and re‐establish cellular homeostasis.

## Conclusions

Transcriptome profiling analysis demonstrated unigene response to dehydration and rehydration in tree peony, namely MYB, AP2/ERF, NAC, bHLH, RING‐H, HSP, and HIPP. These newly identified unigenes will increase our understanding of drought stress–responsive mechanisms, and they may be quite useful as novel genes for the molecular breeding of tree peony to improve its drought tolerance. Further research is necessary to reveal and understand how antioxidant enzymes interact with key hormones in the signaling responses of plants to drought stress.

## Data Accessibility

The cDNA library was deposited in the National Center for Biotechnology Information (NCBI) Transcriptome Shotgun Assembly database (BioSample accession no. SRS1180651).

## Appendix 2 Gene ontology enrichment analysis of biological processes (*P* ≤ 0.05).^a^

**Table:**

Biological process	LYH‐CK vs. LYH‐DR (3785)	LYH‐CK vs. LYH‐RE (2241)	LYH‐DR vs. LYH‐RE (4038)	WLPS‐CK vs. WLPS‐DR (5682)	WLPS‐CK vs. WLPS‐RE (2073)	WLPS‐DR vs. WLPS‐RE (5482)	LYH‐CK vs. WLPS‐CK (2087)	LYH‐DR vs. WLPS‐DR (2614)	LYH‐RE vs. WLPS‐RE (1759)
Oxygen‐containing compound	14.5%	18.3%	14.6%	14.5%	16.4%	14.4%		14.0%	
Oxidation‐reduction process	15.7%	15.7%		15.1%		16.5%	15.1%	16.6%	16.3%
Stimulus	41.7%	45.9%	43.1%	40.9%	43.1%	41.7%			
Abiotic stimulus	19.2%	22.3%	19.7%	19.2%	19.9%	18.8%			
Endogenous stimulus	10.5%	13.4%	11.6%	10.9%	11.9%	10.8%			
Biotic stimulus	10.5%		10.9%	10.2%		10.4%		10.6%	
Chemical stimulus	23.2%	27.0%	23.6%	22.4%		22.9%			
Stress		27.0%							
Organic substance		17.8%	16.1%	15.6%		15.4%			
Single‐organism metabolic process	35.2%		36.6%			35.1%		34.5%	
Single‐organism biosynthetic process	11.8%		11.4%			11.0%			
Single‐organism transport			19.8%	19.1%		18.9%			
Single organism signaling			11.5%	10.8%		11.0%			
Signaling			11.5%	10.8%		11.0%			
Signal transduction			11.2%	10.2%		10.7%			
Cell communication			13.5%	12.8%		13.3%			
Other organism	10.2%					10.0%			
Inorganic substance		11.7%					11.0%		
Organic acid metabolic process				12.8%		12.8%			
Oxoacid metabolic process				12.8%		12.8%			
Carboxylic acid metabolic process				12.7%		12.7%			

Notes

CK = control treatment; DR = drought treatment; LYH = ‘Luo Yang Hong’ cultivar; RE = rehydration treatment; WLPS = ‘Wu Long Peng Sheng’ cultivar.

a Numbers in parentheses in the column headings represent the number of unigenes.

## Appendix 3 Gene ontology enrichment analysis of molecular functions (*P* ≤ 0.05).^a^

**Table:**

Molecular function	LYH‐CK vs. LYH‐DR (3697)	LYH‐CK vs. LYH‐RE (2196)	LYH‐DR vs. LYH‐RE (3996)	WLPS‐CK vs. WLPS‐DR (5522)	WLPS‐CK vs. WLPS‐RE (2017)	WLPS‐DR vs. WLPS‐RE (5340)	LYH‐CK vs. WLPS‐CK (2106)	LYH‐DR vs. WLPS‐DR (2634)	LYH‐RE vs. WLPS‐RE (1792)
Oxidoreductase activity	17.6%	19.3%	19.1%	16.4%	15.9%	18.2%	17.0%	17.6%	18.2%
Catalytic activity	68.8%		70.8%	67.1%		68.1%	69.4%	68.5%	70.3%
Transporter activity	11.5%		2.1%			11.5%			
Protein kinase activity			11.0%			10.4%			
Phosphotransferase activity, alcohol group as acceptor			11.9%			11.5%			
Kinase activity			13.8%						
Transmembrane transporter activity			10.0%						

Note

CK = control treatment; DR = drought treatment; LYH = ‘Luo Yang Hong’ cultivar; RE = rehydration treatment; WLPS = ‘Wu Long Peng Sheng’ cultivar.

a Numbers in parentheses in the column headings represent the number of unigenes.

## Appendix 4 Gene ontology enrichment analysis of cellular components (*P* ≤ 0.05).^a^

**Table:**

Cellular component	LYH‐CK vs. LYH‐DR (3683)	LYH‐CK vs. LYH‐RE (2031)	LYH‐DR vs. LYH‐RE (3800)	WLPS‐CK vs. WLPS‐DR (5503)	WLPS‐CK vs. WLPS‐RE (1879)	WLPS‐DR vs. WLPS‐RE (5235)	LYH‐CK vs. WLPS‐CK (1969)	LYH‐DR vs. WLPS‐DR (2373)	LYH‐RE vs. WLPS‐RE (1609)
Membrane	43.6%	43.2%	48.3%	43.8%	41.6%	46.3%		42.3%	
Cell periphery	27.8%	28.4%	31.0%	27.3%	26.8%	28.2%			
Plasma membrane	22.3%	23.7%	25.7%	23.1%		23.7%			
Extracellular region	10.7%	9.8%	10.9%	9.1%	9.7%	9.2%	9.3%		9.3%
Chloroplast	25.7%								
Plastid	29.2%								

Note

CK = control treatment; DR = drought treatment; LYH = ‘Luo Yang Hong’ cultivar; RE = rehydration treatment; WLPS = ‘Wu Long Peng Sheng’ cultivar.

a Numbers in parentheses in the column headings represent the number of unigenes.
